# Effects of the Gas-Atomization Pressure and Annealing Temperature on the Microstructure and Performance of FeSiBCuNb Nanocrystalline Soft Magnetic Composites

**DOI:** 10.3390/ma16031284

**Published:** 2023-02-02

**Authors:** Guanzhi Li, Guibing Shi, Hongyi Miao, Dan Liu, Zongzhen Li, Mingxu Wang, Li Wang

**Affiliations:** 1 School of Mechanical, Electrical & Information Engineering, Shandong University, Weihai 264209, China; 2 Weihai Wanfeng Magnesium Industry Science and Technology Development Co., Ltd., Weihai 263200, China; 3 Jiangsu JITRI Advanced Energy & Materials Research Institute Co., Ltd., Changzhou 213000, China

**Keywords:** gas atomization, nanocrystalline soft magnetic powders, soft magnetic composites, stress relief

## Abstract

FeSiBCuNb powders prepared by the gas atomization method generally exhibit a wide particle size distribution and a high degree of sphericity. In addition, the correspondingly prepared nanocrystalline soft magnetic composites (NSMCs) perform good service stability. In this paper, effects of the gas-atomization pressure and annealing temperature on the microstructure and soft magnetic properties of FeSiBCuNb powders and NSMCs are investigated. The results show that the powders obtained by a higher gas-atomization pressure possess a larger amorphous ratio and a smaller average crystallite size, which contribute to the better soft magnetic performance of the NSMCs. After being annealed at 550 °C for 60 min, the NSMCs show a much better performance than those treated by the stress-relief annealing process under 300 °C, which indicates that the optimization of the soft magnetic properties resulting from the precipitation of the α-Fe(Si) nanocrystalline largely overwhelms the deterioration caused by the grain growth of the pre-existing crystals. In addition, the annealed NSMCs prepared by the powders with the gas-atomization pressure of 4 MPa show the best performance in this work, *μ*_e_ = 33.32 (*f* = 100 kHz), *H*_c_ = 73.08 A/m and *P*_cv_ = 33.242 mW/cm^3^ (*f* = 100 kHz, *B*_m_ = 20 mT, sine wave).

## 1. Introduction

FeSiBCuNb nanocrystalline soft magnetic composites (NSMCs) show excellent soft magnetic properties, such as a high saturation magnetic induction (*B*_s_) and permeability (*μ*_e_), and low coercivity (*H*_c_) and loss (*P*_cv_). They are also widely used in electronic devices such as sensors, inductors, and transformers [1,2,3]. Compared with flaky powders prepared after melt-spinning and strip breakage, the gas-atomized powders show a slightly lower amorphous ratio, but slightly higher sphericity, wider particle size distribution and lower preparation energy consumption [4]. As the spherical powders are insulation coated easily, the correspondingly prepared NSMCs generally have good stability, and a low eddy current loss (*P*_e_) and magnetostrictive coefficient (*λ*_s_) [5]. Furthermore, the wide particle size distribution of the gas-atomized powders makes them pile up easily, which is beneficial in improving the *μ*_e_ [6].

The parameters of gas atomization play a key role in the preparation of soft magnetic powders with high quality. Shi et al. [7] found that, with the increasing gas-atomization pressure, the gas kinetic energy per unit mass of melt increased, and the average particle size of the obtained FeCrMoNiPBCSi powders were smaller. Gao et al. [8] studied the effects of the melt flow rate, gas-atomization pressure and melt superheat on the AlSi10Mggas-atomization powders. They showed, that with a larger gas-atomization pressure and a higher melt superheat, the melt flow rate tended to be smaller, which led to the smaller average particle size of the powders. Alvarez et al. [9] studied the relationship between the cooling rate and the gas atomization process parameters, such as the droplet temperature, gas temperature and thermal conductivity and particle size of powders. Ciftci et al. [10] found that the gas consumption and the crystalline fraction of the FeCoPSiNb powders could be reduced by using a higher gas temperature. However, there are few studies that focus on the influence of the gas-atomization pressure on the microstructure of FeSiBCuNb powders as well as on the soft magnetic properties of the corresponding NSMCs systematically.

Nanocrystalline soft magnetic materials are generally obtained by annealing amorphous soft magnetic materials at an appropriate temperature. The annealing process also has an effective effect on the soft magnetic properties of nanocrystalline materials [11,12]. Zhao et al. [13] systematically studied effects of the annealing temperature on the *B*_s_ and *H*_c_ of FeSiBCuNbP nanocrystalline powders and the *μ*_e_ and *P*_cv_ of the corresponding NSMCs. The results showed that, when the annealing temperature was between 400 °C and 500 °C, only the α-Fe soft magnetic nanocrystalline phase precipitated, and the NSMCs showed good comprehensive soft magnetic properties. However, when the annealing temperature was 550 °C, the comprehensive soft magnetic properties deteriorated sharply due to the precipitation of the hard magnetic phase. Meng et al. [14] systematically studied the effect of annealing time on the properties of the nanocrystalline Fe_83_Si_4_B_10_P_2_Cu_1_ ribbons. Li et al. [15] studied the effect of annealing time on the properties of the Fe-B-P-C-Cu nanocrystalline alloy. They found that, when the annealing time was too long, the grain size increased excessively although the residual stress could be released, which resulted in the increase of *H*_c_ and the decrease of *μ*_e_. Luo et al. [16] systematically studied the effect of magnetic-field annealing on the properties of FeCoSiBCu amorphous powders. They found that magnetic-field annealing could refine grains by increasing the nucleation rate of nanocrystalline grains, which could improve the soft-magnetic properties. It is worth noting that the cooling rate during the gas atomization process is not high enough. In addition, in most cases, the obtained powders are composed of nanocrystalline and amorphous phases. Thus, both crystallization of the amorphous matrix and grain growth of the nanocrystalline may occur during the annealing process, where the latter is harmful to the soft magnetic properties of the NSMCs. However, there is little research in this regard.

In this work, FeSiBCuNb spherical powders are prepared by the gas atomization method. Effects of the gas-atomization pressure on the microstructure and soft magnetic properties of FeSiBCuNb powders are systematically and quantitatively studied. Then, the corresponding NSMCs are prepared. The effects of the gas-atomized powders with different average particle sizes and amorphous ratios on the soft magnetic properties of FeSiBCuNb NSMCs, as well as effects of the annealing temperature on the soft magnetic properties of the NSMCs, are then investigated.

## 2. Experimental Procedure

### 2.1. Preparation of the Powders

The commercial 1K107 Fe_73.5_Si_13.5_B_9_Cu_1_Nb_3_ bulk alloy is used as raw material. The raw alloy ingot is supplied by Advanced Technology (Bazhou, China) Special Powder Co., ltd. The smelting temperature is 1200 °C. Both the smelting and gas-atomization processes are carried out in an argon atmosphere. The gas-atomization pressures are selected as 2 MPa, 3 MPa and 4 MPa, respectively. Fine powders passed through a 325-mesh sieve are used for the study. For convenience, the powders without annealing treatment are named as P2-raw, P3-raw, and P4-raw. A portion of the powders are annealed at 300 °C or 550 °C. Accordingly, the powders after annealing treatment are named as PX-Y, where X and Y refer to the corresponding gas-atomization pressure and the annealing temperature, respectively.

### 2.2. Preparation and Heat-Treatment of the NSMCs

The fine powders are made into the NSMCs. REN60 silicone resin is selected as the insulating coating agent and adhesive, and acetone is selected as the solvent. Firstly, 3 wt.% REN60 silicone resin is uniformly dispersed in acetone by ultrasonic and mechanical stirring. Secondly, the powders are added to the mixed solution and stirred continuously until the acetone is completely volatilized. Then, the insulated powders are put into the mold after drying in a 60 °C oven for 60 min. The annular NSMCs are obtained under 1400 MPa. The pressurization rate is 360 MPa/min and the holding time is 1 min. Lastly, the NSMCs are cured at 180 °C for 60 min. Similar to the naming method of powders, the NSMCs are named as CX-Y.

### 2.3. Characterization Techniques

The particle size distribution of the powders is tested using a laser particle size analyzer (Bettersize2600, Bettersize Instruments Ltd., Dandong, China). The phase microstructure is analyzed using X-ray diffraction (Rigaku D/max-rB, Cu Kα). Differential scanning calorimetry (TA Instruments, New Castle, DE, USA) is used to characterize the crystallization behavior of powders with a heating rate of 40 °C/min. A scanning electron microscope (Hitachi Regulus 8100, Chiyoda, Japan) is used to test the morphology of the powders. A soft magnetic direct current measuring instrument (DSMC-8200SD, Loudi, China) is used to test the *H*_c_ of the NSMCs. The *μ*_e_ and DC-bias performance of the NSMCs are tested by a Lenz capacitor resistance meter (LCR-8210, Taiwan, China, 10 kHz~1 MHz). The *P*_cv_ of the NSMCs is measured by a soft magnetic alternating current analyzer (MAST-3000SA, Loudi, China, 10 kHz~1 MHz) at 20 mT.

## 3. Results and Discussion

### 3.1. Structure and Soft-Magnetic Properties of the Gas-Atomized Powders

**Figure 1:**
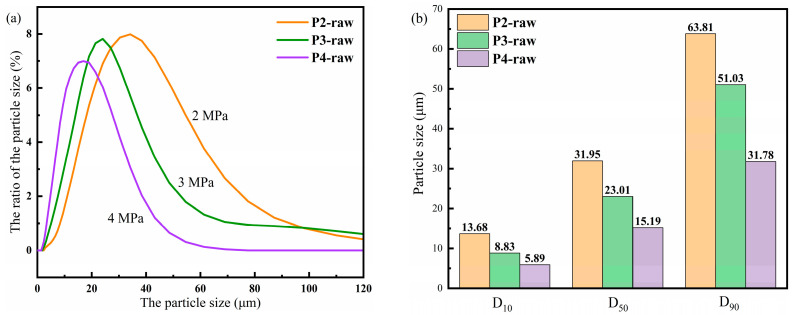
The particle size distribution (**a**) and the symbolic size (**b**) of the powders with different gas-atomization pressures.

The particle size distribution and the symbolic size of the powders with different gas-atomization pressures are shown in Figure 1. One sees from Figure 1 that the FeSiBCuNb powders prepared at a higher gas-atomization pressure not only are finer, but also show a narrower particle size distribution. With the increasing gas-atomization pressure, the gas kinetic energy per unit mass of melt becomes higher. As a result, the crushing of melt is more sufficient, and the average particle size of the powders is finer. P4-raw is shown to be the finest powder, with a D_50_ = 15.19 μm.

**Figure 2:**
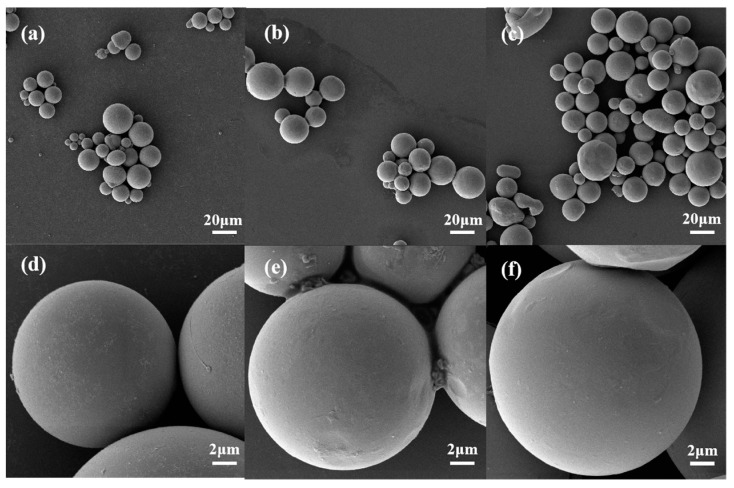
The SEM images of the powders. (**a**) Low magnification of P2-raw, (**b**) low magnification of P3-raw, (**c**) low magnification of P4-raw, (**d**) high magnification of P2-raw, (**e**) high magnification of P3-raw, (**f**) high magnification of P4-raw.

All the SEM images of the powders are shown in Figure 2, where the SEM images for all of the raw powders made by different gas-atomization pressures with both low and high magnifications are supplemented. The SEM images of P2-raw, P3-raw, and P4-raw at low magnification are shown in Figure 2a–c, respectively, while the corresponding images with high magnification are shown in Figure 2d–f, respectively. One sees from Figure 2 that the powders possess excellent sphericity and a smooth surface, which indicates that the powders can easily be coated, and the correspondingly prepared NSMCs should have good stability and relatively low loss. Moreover, there are almost no satellite powders around.

**Figure 3:**
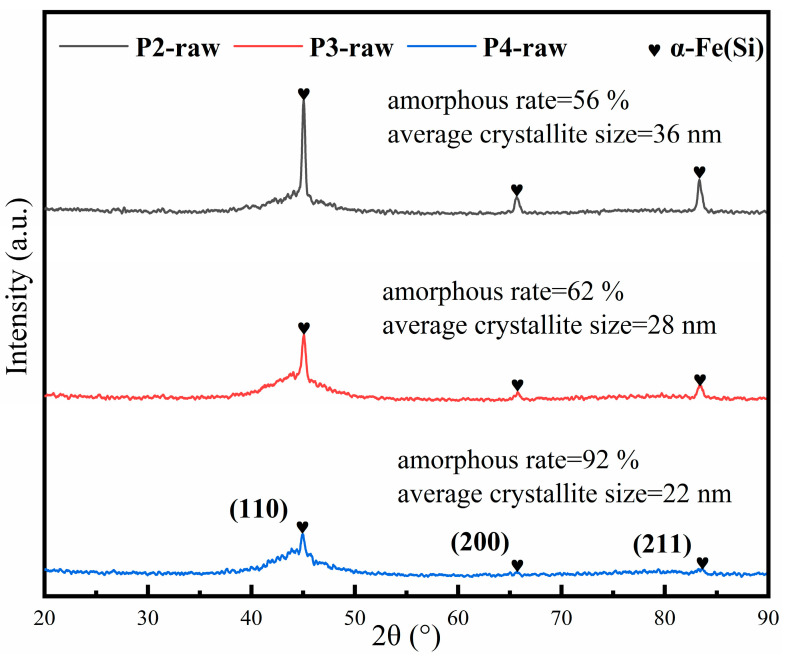
The XRD patterns of the powders without annealing treatment.

To identify the contained phases in the FeSiBCuNb powders, the XRD patterns are shown in Figure 3. One sees from Figure 3 that there is a broad diffuse peak corresponding to the amorphous phase, while sharp peaks corresponding to the (110), (200), and (211) crystal planes of α-Fe (Si) appear distinctly at 2θ = 44.8°, 65.3°, and 82.7°, respectively [13]. With the increasing gas-atomization pressure, the intensity of these three peaks decreases, indicating the decrease of the crystalline ratio. In addition, when the pressure is 4 MPa, there is only a very small diffraction peak at 2θ = 44.8°. To quantify the results of the XRD pattern, both the volume fraction of the amorphous phase (*V*_am_) and the average crystallite size (*d*) are estimated according to Equation (1) [17] and Equation (2) [18], respectively.

(1) $$V_{am}=\frac{I_{am}}{I_{cr}+I_{am}}$$

(2) $$d=\frac{K\lambda }{\beta \cos\theta }$$

where *I*_am_ and *I*_cr_ are integral intensities of the diffraction peaks of the amorphous phase and crystalline phase, respectively, *K* is the shape factor equal to 0.94, *λ* is the wave-length of the X-ray used, *β* is the full width at half maxima, and *θ* is the angle between the incident and the scattered X-ray. The calculated *V*_am_ values of P2-raw, P3-raw, and P4-raw are about 56%, 62%, and 92%, respectively, while the estimated *d* are 36 nm, 28 nm and 22 nm, correspondingly.

**Figure 4:**
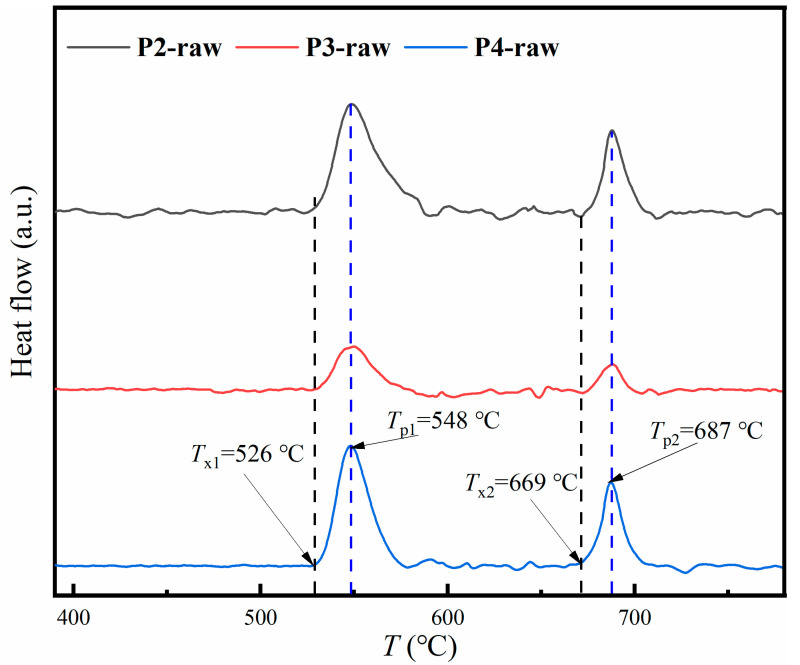
The DSC curves of the powders at a heating rate of 40 °C/min.

In order to find the proper annealing temperature, the crystallization behavior of the powders is shown in the DSC curves in Figure 4. One sees that there are two peaks in each DSC curve. The first exothermic peak corresponds to the precipitation of the α-Fe (Si) phase, while the second peak corresponds to the hard-magnetic phase, including the Fe-boron phases [19,20,21,22,23]. It can be seen that the gas-atomization pressure has little effect on the onset temperatures (*T*_x1_, *T*_x2_) and the peak temperatures (*T*_p1_, *T*_p2_) for both the primary process and the secondary crystallization, as the position of the peaks barely changes with the increasing gas-atomization pressure. Similarly, the gas-atomization pressure does not effectively affect the thermal stability of the powders. Furthermore, the temperature intervals between two peaks for all the powders are relatively large (>135 °C), indicating the good nanocrystallization-forming ability of the FeSiBCuNb system to some extent [24,25].

**Figure 5:**
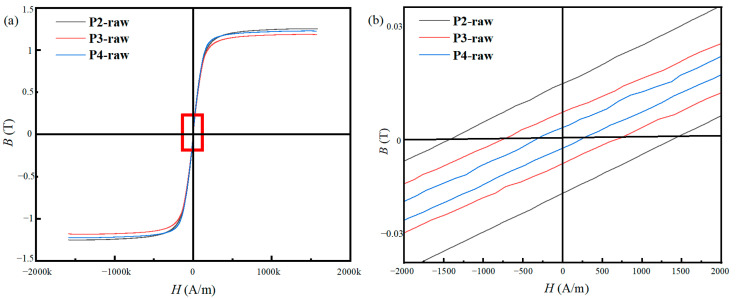
The hysteresis loops of the powders (**a**) and the partial enlarged detail (**b**).

The hysteresis loops of the powders are shown in Figure 5. It is well known that the microstructure, noticeably the crystallite size, essentially determines the hysteresis loop of a ferromagnetic material. While *d* is lower than 100 nm, the *H*_c_ of the powders is positively correlated with *d*^6^ [26]. One sees that the *H*_c_ of P2-raw, P3-raw, and P4-raw are 1407.1 A/m, 721.9 A/m, and 288.2 A/m, respectively. The *H*_c_ of materials can be simply represented as follows [27]:

(3) $$H_{c}=\frac{P_{c}K_{1}d^{6}}{J_{s}A^{3}}$$

where *P*_c_ is a constant, *J*_s_ is the saturation magnetization, *K*_1_ is the magneto-crystalline anisotropy constant, and *A* is the ferromagnetic exchange constant between adjacent grains. As mentioned above, the powders prepared with a higher gas-atomization pressure show a smaller *d*. Thus, according to Equation (3), the *H*_c_ of the powders should decrease sharply with the increase of the gas-atomization pressure, which is in accordance with the results shown in Figure 5. The *B*_s_ of P2-raw, P3-raw, and P4-raw are 1.25 T, 1.18 T, and 1.22 T, respectively. There is no significant association between the gas-atomization pressure and *B*_s_, since *B*_s_ is mainly related to the content of the ferromagnetic elements.

### 3.2. Soft-Magnetic Properties of the NSMCs

**Figure 6:**
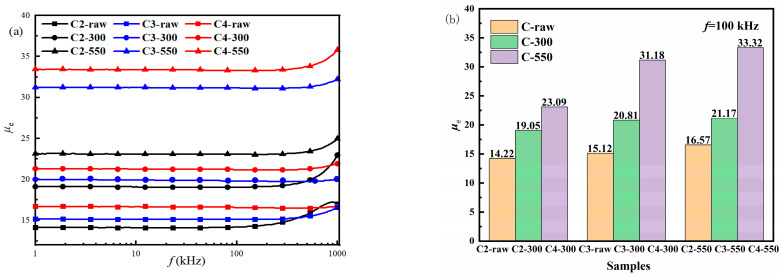
The frequency dependent characteristics of *μ*_e_ (**a**) and the comparison of *μ*_e_ (*f* = 100 kHz) (**b**) of the NSMCs.

After preparing the gas-atomized powders into the NSMCs, the effects of the annealing temperature on the soft magnetic properties of the NSMCs are investigated. The dependence of frequency (*f*) on *μ*_e_ for the NSMCs is shown Figure 6a. One sees that all the samples exhibit excellent high-frequency stability of the *μ*_e_, which keeps at a constant value below 100 kHz. One sees from Figure 6 that, after the heat treatment, the *μ*_e_ of FeSiBCuNb NSMCs prepared by the gas-atomized powder with a higher gas-atomization pressure is shown to be larger. The *μ*_e_ of FeSiBCuNb NSMCs also increases with the increasing annealing temperature. Figure 6b shows the *μ*_e_ of the NSMCs at *f* = 100 kHz. Among all the samples, the C4-550 has the highest *μ*_e_, which is 33.32 (*f* = 100 kHz).

As is known, *μ*_e_ is closely related to residual stresses, defects, and the content of non-magnetic materials, [28] which can be expressed by Equation (4).

(4) $$\mu_{e}=\frac{B_{s}{}^{2}}{aK_{1}+b\lambda_{s}\sigma }=\frac{\mu^{\prime }d}{\mu^{\prime }c+d}$$

where *a* and *b* are constants, σ is the residual stress, $\mu^{\prime }$ is the intrinsic permeability of the soft magnetic powders, and *c* is the non-magnetic material content. The non-magnetic material content mainly includes the air gaps and defects between powders during forming and the organic insulating coating agent, since these materials contain no magnetic elements. Generally, the *c* is mainly related to the electrical resistivity of the NSMCs.

According to Equation (4), the *μ*_e_ of the FeSiBCuNb NSMCs with a smaller *d* should be higher, which is in accordance with the results shown in Figure 3 and Figure 6. Meanwhile, as the powders under a higher gas-atomization pressure show a smaller average particle size (see Figure 1), they pile up more easily. Thus, there is less air gap in the NSMCs made by the finer powders, which reduces the *c* and then causes the increase of *μ*_e_ to some extent. In other words, under the same annealing treatment, the higher *μ*_e_ of FeSiBCuNb NSMCs made by the powders with a higher gas-atomization pressure should be attributed to the lower *d* and *c*.

**Figure 7:**
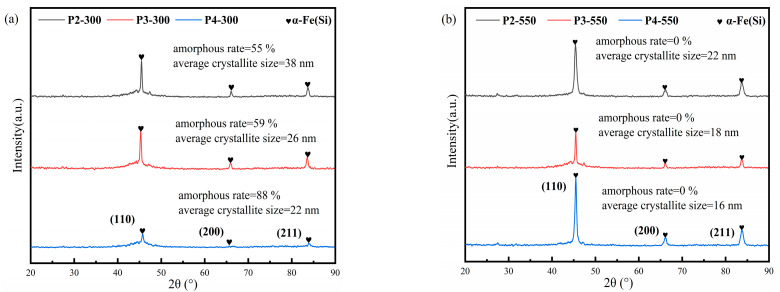
The XRD patterns of the powders after being annealed at 300 °C (**a**) and 550 °C (**b**).

In order to discover the effect of annealing treatment on the *d* of the NSMCs as well as to exclude the effect introduced by the insulation coating agent, the XRD patterns of the gas-atomized powders with different annealing treatments are shown in Figure 6. Comparing Figure 7a with Figure 3, one sees that the amorphous ratio and the *d* of the powders barely change after being annealed at 300 °C, where the slightly decreased amorphous ratio and slightly increased *d* result from the grain growth of the pre-exist nanocrystalline in the amorphous matrix. However, comparing Figure 7a with Figure 7b, one sees that the amorphous ratio decreases to 0% and the *d* decreases sharply for all the powders after being annealed at 550 °C, which indicates that large amounts of α-Fe (Si) nanocrystalline grains with a smaller *d* have precipitated from the amorphous matrix, though further grain growth of the pre-existing nanocrystalline is also promoted.

It is known that annealing at an appropriate temperature can not only release the residual stress caused by crystallization and cold pressing, but also promote the precipitation of nanocrystalline. Relief of the residual stress in the NSMCs reduces the pinning effect and the magnetic anisotropy, which can reduce *λ*_s_ and then improve *μ*_e_ [10], while the precipitation of nanocrystalline effectively increases the *μ*_e_, as *λ*_s_ of the α-Fe (Si) nanocrystalline is lower than that of the amorphous matrix. Thus, it can be speculated that the increased *μ*_e_ of the NSMCs after being annealed at 300 °C should mainly result from the relief of the residual stress, since both the amorphous ratio and the *d* barely change compared with those without annealing treatment. Comparing those after annealing at 300 °C, the largely increased *μ*_e_ of the NSMCs after annealing at 550 °C should be mainly attributed to the obviously decreased *d* of the gas-atomized FeSiBCuNb powders, according to Equation (4). Furthermore, with the increasing annealing temperature, the evaporation of the insulation coating agent causes the reduction of *c* to some extent, which also leads to the increase of *μ*_e_.

**Figure 8:**
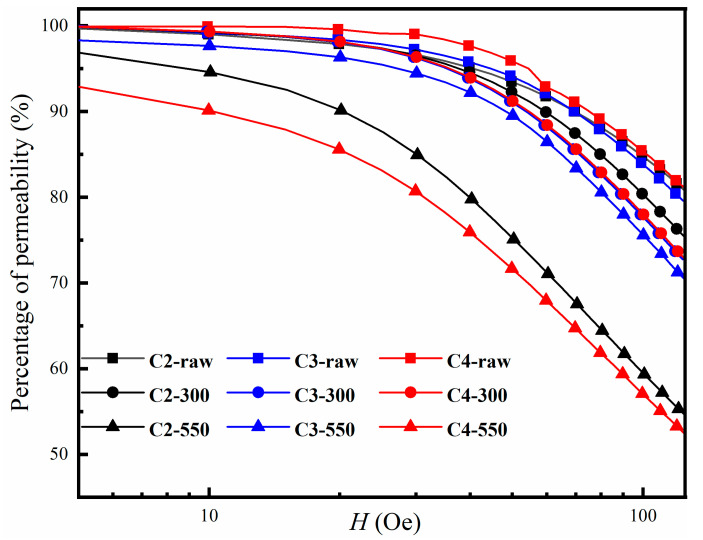
The DC-bias characteristics of the NSMCs.

The DC-bias performance of the NSMCs is shown in Figure 8. One sees that the NSMCs made by the powders with a higher gas-atomization pressure show a lower DC-bias performance. However, the DC-bias performance worsens with the increasing annealing temperature, which is opposite to the trend for *μ*_e,_ as the higher the *μ*_e_ is, the greater the magnetic induction intensity of the NSMCs will be under the same applied magnetic field. In turn, it is easier for the magnetic induction to reach saturation. When the applied magnetic field is 100 Oe without annealing, the best DC-bias performance in this study can reach 85%, corresponding to C2-raw (see Figure 8), while the worst is about 57%, as the precipitation of the nanocrystals in C4-550 tends to largely increase the *μ*_e_. The NSMCs with excellent DC-bias performance can be widely used in a high current field.

**Figure 9:**
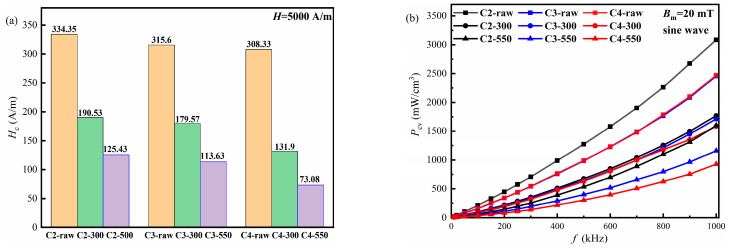
The frequency dependent characteristics of *H*_c_ (**a**) and the *P*_cv_ (**b**) of the NSMCs.

The dependence of the frequency on the *H*_c_ and the *P*_cv_ of the NSMCs is shown in Figure 9a,b, respectively. One sees from Figure 9 that a similar trend can be found between *H*_c_ and *P*_cv_. In addition, both the *H*_c_ and *P*_cv_ of the NSMCs prepared by the powders with a higher gas-atomization pressure are shown to be smaller under the same annealing treatment. Compared with those without heat treatment, the *P*_cv_ of FeSiBCuNb NSMCs decreases after being annealed at 300 °C, and further decreases after being annealed at 550 °C. As is known, *P*_cv_ includes hysteresis loss (*P*_h_), *P*_e_ and residual loss (*P*_r_) [29]. In addition, *P*_r_ generally results from magnetization relaxation and resonance of the domain walls, which can be ignored in most cases [30]. Thus, the *P*_cv_ of the NSMCs can be represented as follows:

(5) $$P_{\mathrm{cv}}=P_{h}+P_{e}=K_{h}B_{m}^{1.6}f+K_{e}B_{m}^{2}f^{2}$$

where *K*_h_ and *K*_e_ are the hysteresis loss coefficient and the eddy current loss coefficient, respectively, and *B*_m_ is the maximum magnetic induction strength. Due to the presence of the insulation coating, the *P*_e_ of the NSMCs is relatively small. As a result, the *P*_h_ is dominant for the *P*_cv_ of the NSMCs, and the *K*_h_ or *P*_h_ is proven to be positively correlated with the *H*_c_ of the NSMCs. According to Equation (3), The *H*_c_ decreases with the decreasing *d*. Thus, under the same annealing treatment, the lower *P*_cv_ of the FeSiBCuNb NSMCs made by the powders with a higher gas-atomization pressure should be attributed to the lower *H*_c_ of the raw powders, as these powders show a smaller *d* and higher *V*_am_. Compared with the NSMCs without annealing, both *H*_c_ and *P*_cv_ of the NSMCs decrease after being annealed at 300 °C, and decrease more after being annealed at 550 °C. As mentioned previously, the stress-relief annealing treatment at 300 °C only has a slight effect on grain growth but largely reduces the residual stress in the NSMCs, which leads to the reduction of the *K*_h_ and *H*_c_ of the NSMCs. Meanwhile, the nanocrystallization annealing treatment under 550 °C can not only further reduce the residual stress but also promote the precipitation of α-Fe (Si) nanocrystals from the amorphous matrix, which in turn further decreases the *H*_c_ and *P*_cv_.

## 4. Conclusions

In this work, effects of the gas-atomization pressure on the size distribution, microstructure and magnetic properties of FeSiBCuNb powders, as well as the effects of annealing temperature on the soft magnetic properties of the corresponding NSMCs, are systematically studied. The following conclusions are as follows:

(1) The obtained powders contain the amorphous phase and the α-Fe (Si) phase. With the increasing gas-atomization pressure, the soft-magnetic properties of the corresponding powders and NSMCs tend to be better, which can be attributed to the smaller *d* and the larger amorphous ratio of the powders. The powders prepared by the 4 MPa gas-atomization pressure without annealing treatment show the highest amorphous ratio of 92% in this study.
(2) After annealing treatment, the *μ*_e_ of NSMCs increases compared with the raw NSMCs, while *H*_c_ and *P*_cv_ decrease. In addition, the nanocrystallization annealing treatment at 550 °C can largely optimize the soft magnetic properties of the NSMCs, which is better than the stress-relief annealing treatment at 300 °C. It can thus be suspected that the improvement of soft magnetic properties resulting from the precipitation of the α-Fe(Si) nanocrystals largely overwhelms the deterioration caused by the grain growth.
(3) After being annealed at 550 °C, the NSMCs made by the powders using the 4 MPa gas-atomization pressure show the best performance among this work with *μ*_e_ = 33.32 (*f* = 100 kHz), *H*_c_ = 73.08 A/m, and *P*_cv_ = 33.242 mW/cm^3^ (*f* = 100 kHz, *B*_m_ = 20 mT, sine wave), resulting from the stress relief and the smallest *d* of the precipitated α-Fe (Si) nanocrystalline.

## Data Availability

All data included in this study are available upon request by contact with the corresponding author.

## References

[1] Han Y., Wei R., Li Z., Li F., Wang A., Chang C., Wang X. (2017). Improvement of magnetic properties for V-substituted Fe_73.5_Si_13.5_B_9_Cu_1_Nb_3-x_V_x_ nanocrystalline alloys. J. Mater. Sci..

[2] Gao S., Yan X., Chang C., Aubry E., He P., Liu M., Liao H., Nouredine F. (2021). Microstructure and magnetic properties of FeSiBCrC soft magnetic alloy manufactured by selective laser melting. Mater. Lett..

[3] Xiao Z., Tang C., Zhao H., Zhang D., Li Y. (2012). Effects of sintering temperature on microstructure and property evolution of Fe_81_Cu_2_Nb_3_Si_14_ soft magnetic materials fabricated from amorphous melt-spun ribbons by spark plasma sintering technique. J. Non. Cryst. Solids..

[4] Liu M., Liu L., Cai P., Dong Y., Wang X. (2018). Fabrication and magnetic properties of novel Fe-based amorphous powder and corresponding powder cores. J. Mater. Sci. Mater. Electron..

[5] Li Z., Dong Y., Pauly S., Chang C., Wei R., Li F., Wang X. (2017). Enhanced soft magnetic properties of Fe-based amorphous powder cores by longitude magnetic field annealing. J. Alloys Compd..

[6] Anhalt M. (2008). Systematic investigation of particle size dependence of magneticproperties in soft magnetic composites. J. Magn. Magn. Mater..

[7] Shi Y., Lu W., Sun W., Zhang S., Yang B., Wang J. (2022). Pressure-dependent microstructure evolution of Fe-based amorphous alloy powders via high-pressure gas atomization. J. Alloys Compd..

[8] Gao C., Xiao Z., Zou H., Liu Z., Chen J., Li S., Zhang D. (2019). Characterization of spherical AlSi10Mg powder produced by double-nozzle gas atomization using different parameters. T. Nonferr. Metal Soc..

[9] Alvarez K.L., Martin J.M., Lpatov M., Gonzalez J. (2017). Soft magnetic amorphous alloys (Fe-rich) obtained by gas atomisation technique. J. Alloys Compd..

[10] Ciftci N., Ellendt N., Barreto E.S., Mädler L., Uhlenwinkel V. (2018). Increasing the amorphous yield of {(Fe_0.6_Co_0.4_)_(0.75)_B_0.2_Si_0.05_}_(96)_Nb_4_ powders by hot gas atomization. Adv. Powder Technol..

[11] Cao C., Wang Y., Zhu L., Meng Y., Dai Y., Chen J. (2017). Evolution of structural and magnetic properties of the FeCuBP amorphous alloy during annealing. J. Alloys Compd..

[12] Wang J., Liu X., Zheng Z., Qiu Z., Li K., Xu J., Lu K., Zeng D. (2022). Reduction of core loss for FeSi soft magnetic composites prepared using atomic layer deposition-based coating and high-temperature annealing. J. Alloys Compd..

[13] Zhao T., Yu H., Sun C., Chen C., Hao J. (2022). Effects of the substitution of Si with P on the glass forming ability, crystallization behavior, and magnetic properties of FeCuNbSiBP atomized powder. J. Magn. Magn. Mater..

[14] Meng Y., Pang S., Chang C., Bai X., Zhang T. (2021). Nanocrystalline Fe_83_Si_4_B_10_P_2_Cu_1_ ribbons with improved soft magnetic properties and bendability prepared via rapid annealing of the amorphous precursor. J. Magn. Magn. Mater..

[15] Li Y., Shen N., Wu Y., Zhang S., He Z., Li F., Hui X. (2022). High B_s_ Fe-B-P-C-Cu nanocrystalline alloy with longtime annealing stability and low heating rate sensitivity. J. Magn. Magn. Mater..

[16] Luo Q., Li D., Cai M., Di S., Zhang Z., Zeng Q., Wang Q., Shen B. (2022). Excellent magnetic softness-magnetization synergy and suppressed defect activation in soft magnetic amorphous alloys by magnetic field annealing. J. Mater. Sci. Technol..

[17] Xing Y., Dong B., Zhou S., Dong Y., Chen W., Cui H., Wang L., Wang J. (2022). Soft magnetic properties of Co-doped FeSiBC amorphous and nanocrystalline alloys. J. Magn. Magn. Mater..

[18] Yadav K., Sangwan R., Barala M., Mohan D., Sanghi S. (2022). Effect of antimony dopant on the structural properties of CdSe crystalline chalcogenides. Mater. Today Proc..

[19] Henzer G. (1997). Soft magnetic nanocrystalline materials. Handb. Magn. Mater..

[20] Li Y., Dou Z., Chen X., Lv K., Li F., Hui X. (2020). Improving the amorphous forming ability and magnetic properties of FeSiBPCu amorphous and nanocrystalline alloys by utilizing carbon. J. Alloys Compd..

[21] Shi L., Yao K. (2020). Composition design for Fe-based soft magnetic amorphous and nanocrystalline alloys with high Fe content. Mater. Des..

[22] Wu C., Chen H., Lv H., Yan M. (2016). Interplay of crystallization, stress relaxation and magnetic properties for FeCuNbSiB soft magnetic composites. J. Alloys Compd..

[23] Atnami H., Grognet S., Teillet J. (2001). Crystallization-nitriding process of FeSiB and FeSiBCuNb ribbons: Influence of additive (Cu, Nb) pair and nitrogen on structure, magnetic and magnetostrictive parameters. J. Mater. Sci..

[24] Lee S., Kato H., Kubota T., Makino A., Inoue A. (2009). Fabrication and soft magnetic properties of Fe–B–Nb–Y glassy powder compacts by spark plasma sintering technique. Intermetallics.

[25] Li X., Liu J., Qu C., Song K., Wang L. (2017). Effects of Nb on the precipitation of α-Fe, glass forming ability and magnetic properties of Fe_85_B_10_P_5_ alloys. J. Alloys Compd..

[26] Herzer G. (1995). Soft magnetic nanocrystalline materials. Scr. Mater..

[27] Tian M., Xu J., Yang S., Wang J., Yang T., Li G., Chen Q., Liu X. (2022). Effects of heat treatment and compaction pressure on the microstructure and magnetic properties of core-shell structured FeSiBNbCu/SiO_2_ soft magnetic composites. J. Alloys Compd..

[28] Korvánek I., Kim C.G., Kováč J., Švec P., Sato-Turtelli R. (2000). Soft magnetic behaviour and permeability spectra in amorphous and nanocrystalline Fe_80.5_Nb_7_B_12.5_ alloys. J. Magn. Magn. Mater..

[29] Zhao R., Huang J., Yang Y., Jiao L., Dong Y., Liu X., Liu Z., Wu S., Li X., He A. (2022). The influence of FeNi nanoparticles on the microstructures and soft magnetic properties of FeSi soft magnetic composites. Adv. Powder Technol..

[30] Li X., Dong Y., Wu S., Zhao R., Ding Q., Jia X., He A., Li J., Liu X. (2022). Evolution of magnetic domain structure and magnetic properties of Fe-based nanocrystalline powder cores during transverse magnetic field annealing. Adv. Powder Technol..

